# Supplementary material for: Developmental NMDA receptor dysregulation in the infantile neuronal ceroid lipofuscinosis mouse model
Source: eLife. 2019 Apr 4;8:e40316. doi: 10.7554/eLife.40316 (PMC6464704; doi:10.7554/eLife.40316)
Supplement: Supplementary file 1. — Values are represented for each layer, at each age in WT and Ppt1-/- mice. [file elife-40316-supp1.docx]

|  | Layer | WT | | | Mutant | | |
| --- | --- | --- | --- | --- | --- | --- | --- |
|  |  | **Mean** | **SEM** | **n** | **Mean** | **SEM** | **n** |
| P11 | **1** | 0.014726 | 0.051334 | 4 | 0.018432 | 0.020171 | 5 |
|  | **2/3** | 0.014029 | 0.002 |  | 0.015746 | 0.001837 |  |
|  | **4** | 0.017716 | 0.005554 |  | 0.018337 | 0.003265 |  |
|  | **5** | 0.013956 | 0.003186 |  | 0.017544 | 0.002825 |  |
|  | **6** | 0.016376 | 0.004648 |  | 0.015585 | 0.009563 |  |
| P14 | **1** | 0.020027 | 0.003256 | 3 | 0.032299 | 0.003201 | 6 |
|  | **2/3** | 0.012068 | 0.003681 |  | 0.026184 | 0.003002 |  |
|  | **4** | 0.011678 | 0.001782 |  | 0.03401 | 0.004257 |  |
|  | **5** | 0.011427 | 0.003523 |  | 0.028279 | 0.003798 |  |
|  | **6** | 0.014201 | 0.004393 |  | 0.024822 | 0.008465 |  |
| P28 | **1** | 0.025066 | 0.030382 | 4 | 0.054899 | 0.050557 | 5 |
|  | **2/3** | 0.032533 | 0.008919 |  | 0.111296 | 0.020856 |  |
|  | **4** | 0.030768 | 0.01448 |  | 0.125996 | 0.021164 |  |
|  | **5** | 0.021373 | 0.004357 |  | 0.113626 | 0.017283 |  |
|  | **6** | 0.021647 | 0.007201 |  | 0.086127 | 0.013937 |  |
| P33 | **1** | 0.028305 | 0.073043 | 5 | 0.117601 | 0.023632 | 6 |
|  | **2/3** | 0.022777 | 0.008171 |  | 0.21026 | 0.013193 |  |
|  | **4** | 0.035548 | 0.011861 |  | 0.242556 | 0.034924 |  |
|  | **5** | 0.035123 | 0.010396 |  | 0.197036 | 0.074042 |  |
|  | **6** | 0.048262 | 0.010111 |  | 0.19617 | 0.024701 |  |
| P42 | **1** | 0.019019 | 0.060801 | 4 | 0.113172 | 0.012468 | 5 |
|  | **2/3** | 0.030153 | 0.011804 |  | 0.237298 | 0.021055 |  |
|  | **4** | 0.041546 | 0.011364 |  | 0.253448 | 0.029969 |  |
|  | **5** | 0.042801 | 0.009777 |  | 0.265477 | 0.041234 |  |
|  | **6** | 0.041961 | 0.00768 |  | 0.209104 | 0.035667 |  |
| P60 | **1** | 0.037639 | 0.008034 | 4 | 0.227769 | 0.053888 | 6 |
|  | **2/3** | 0.029154 | 0.009176 |  | 0.297558 | 0.059318 |  |
|  | **4** | 0.046682 | 0.014308 |  | 0.263665 | 0.052311 |  |
|  | **5** | 0.049726 | 0.007089 |  | 0.234832 | 0.038626 |  |
|  | **6** | 0.056535 | 0.011388 |  | 0.197509 | 0.041092 |  |
| P78 | **1** | 0.020962 | 0.005615 | 3 | 0.188845 | 0.002442 | 3 |
|  | **2/3** | 0.020522 | 0.001178 |  | 0.415522 | 0.007618 |  |
|  | **4** | 0.043183 | 0.012138 |  | 0.308193 | 0.037127 |  |
|  | **5** | 0.055858 | 0.004104 |  | 0.205356 | 0.055491 |  |
|  | **6** | 0.045009 | 0.004334 |  | 0.184726 | 0.003858 |  |
| P120 | **1** | 0.046265 | 0.023688 | 3 | 0.32851 | 0.141037 | 3 |
|  | **2/3** | 0.053516 | 0.029019 |  | 0.378299 | 0.065604 |  |
|  | **4** | 0.077311 | 0.038016 |  | 0.349415 | 0.062099 |  |
|  | **5** | 0.058412 | 0.038213 |  | 0.35062 | 0.059149 |  |
|  | **6** | 0.059228 | 0.040012 |  | 0.271503 | 0.068553 |  |
