# Supplementary material for: Developmental NMDA receptor dysregulation in the infantile neuronal ceroid lipofuscinosis mouse model
Source: eLife. 2019 Apr 4;8:e40316. doi: 10.7554/eLife.40316 (PMC6464704; doi:10.7554/eLife.40316)
Supplement: Transparent reporting form [file elife-40316-transrepform.docx]

***eLife’s* transparent reporting form**

We encourage authors to provide detailed information *within their submission* to facilitate the interpretation and replication of experiments. Authors can upload supporting documentation to indicate the use of appropriate reporting guidelines for health-related research (see [EQUATOR Network](http://www.equator-network.org/%20)), life science research (see the [BioSharing Information Resource](https://biosharing.org/)), or the [ARRIVE guidelines](http://www.plosbiology.org/article/info:doi/10.1371/journal.pbio.1000412) for reporting work involving animal research. Where applicable, authors should refer to any relevant reporting standards documents in this form.

If you have any questions, please consult our Journal Policies and/or contact us: [editorial@elifesciences.org](mailto:editorial@elifesciences.org).

**Sample-size estimation**

- You should state whether an appropriate sample size was computed when the study was being designed
- You should state the statistical method of sample size computation and any required assumptions
- If no explicit power analysis was used, you should describe how you decided what sample (replicate) size (number) to use

Please outline where this information can be found within the submission (e.g., sections or figure legends), or explain why this information doesn’t apply to your submission:

For electrophysiological experiments, the power analysis was performed on preliminary studies with G*Power 3, based on a two-tailed t-test between two independent groups, applying 0.80 as desirable power, and alpha equal to 0.05. A group size of 10 cells yields a very high actual power to detect significant changes in the proposed electrophysiological studies. For biochemical/imaging data, our preliminary results as well as the literature using the same mouse model (e.g. PMID: 18704195) informed our samples size selection. To reduce inter-experiment variability, experiments were performed with technical replicates that were subsequently averaged for each sample (n). In developmental neuroscience, examining a minimum of three different animals and repletion of three experiments from a set of samples are typically required for biochemical and anatomical analyses.

**Replicates**

- You should report how often each experiment was performed
- You should include a definition of biological versus technical replication
- The data obtained should be provided and sufficient information should be provided to indicate the number of independent biological and/or technical replicates
- If you encountered any outliers, you should describe how these were handled
- Criteria for exclusion/inclusion of data should be clearly stated
- High-throughput sequence data should be uploaded before submission, with a private link for reviewers provided (these are available from both GEO and ArrayExpress)

Please outline where this information can be found within the submission (e.g., sections or figure legends), or explain why this information doesn’t apply to your submission:

We have included all relevant details of experiment content, data acquisition, and analysis in the Methods section of the manuscript. Further, we have made a point to mention how we represented our sample sizes, including how we categorized technical and biological replicates, within the figure legends and methods section for each experiment. Our responses to the remaining points (3-6) are as follows:

Biological replicates were taken as: *in vivo-* each animal is a biological replicate with exception of *in utero* electroporation data (Figure 4, in which each cell was considered a biological replicate). *In vitro-*with exception of calcium imaging data (Figure 7, where each synaptic site was considered a biological replicate), each independent culture (derived from an independent embryo) is taken as a biological replicate.

Technical replicates are defined as either 1) analysis of repetitions within a single experiment (For example, 8-10 cells/culture were averaged to make up each n in Figure 9 lipofuscin/lysosome data, Figure 9C-E) or 2) repetition of the same experiment, using the same samples, performed distinctly from the original experiment (For example, for immunoblot data same samples run two times). Technical replicates were used/ran wherever possible.

No outliers were encountered and no data were excluded.

**Statistical reporting**

- Statistical analysis methods should be described and justified
- Raw data should be presented in figures whenever informative to do so (typically when N per group is less than 10)
- For each experiment, you should identify the statistical tests used, exact values of N, definitions of center, methods of multiple test correction, and dispersion and precision measures (e.g., mean, median, SD, SEM, confidence intervals; and, for the major substantive results, a measure of effect size (e.g., Pearson's r, Cohen's d)
- Report exact p-values wherever possible alongside the summary statistics and 95% confidence intervals. These should be reported for all key questions and not only when the p-value is less than 0.05.

Please outline where this information can be found within the submission (e.g., sections or figure legends), or explain why this information doesn’t apply to your submission:

Raw data is presented in scatter plot format in the majority of figures, where the authors felt that the data would be best consumed in this way or where the number of comparisons was small enough so that the figure would maintain readability in scatter format. The authors recognize this is not the case for some figures, though we are open to amending the format to represent the raw data in scatter format for these panels as well. Source data was included for Figure 1.

Samples sizes were included in detail for each Figure, in each Figure legend. Statistical tests were also described in the figure legends. Student’s t-tests were used for the majority of experiments, as the experimental design intended to capture differences between the WT and *Ppt1-/-* groups within each experiment, at each time point (or for each measure).

Exact p values are reported for Figures 1 and 8, though the authors are happy to report this data for the other Figures. We apologize for the inconvenience of not reporting this in the full submission manuscript. If necessary, we will submit a table
(as suggested below).

(For large datasets, or papers with a very large number of statistical tests, you may upload a single table file with tests, Ns, etc., with reference to sections in the manuscript.)

**Group allocation**

- Indicate how samples were allocated into experimental groups (in the case of clinical studies, please specify allocation to treatment method); if randomization was used, please also state if restricted randomization was applied
- Indicate if masking was used during group allocation, data collection and/or data analysis

Please outline where this information can be found within the submission (e.g., sections or figure legends), or explain why this information doesn’t apply to your submission:

This information is included in the Materials and Methods section under the heading “Animals, group allocation, and data handling.”

**Additional data files (“source data”)**

- We encourage you to upload relevant additional data files, such as numerical data that are represented as a graph in a figure, or as a summary table
- Where provided, these should be in the most useful format, and they can be uploaded as “Source data” files linked to a main figure or table
- Include model definition files including the full list of parameters used
- Include code used for data analysis (e.g., R, MatLab)
- Avoid stating that data files are “available upon request”

Please indicate the figures or tables for which source data files have been provided:

Source data has been provided for Figure 1, where the scatter plot data is not represented. See abve note in “statistical reporting” section.

Code for data analysis in Figure 7 is included in Methods.
